# Vacuoles, E1 Enzyme, X-linked, Autoinflammatory, Somatic (VEXAS) Syndrome Mimicking Antineutrophil Cytoplasmic Antibody (ANCA)-Associated Vasculitis: A Delayed Diagnosis in a Patient With Multisystem Inflammation and Neutrophilic Dermatosis

**DOI:** 10.7759/cureus.110208

**Published:** 2026-06-03

**Authors:** Katherine E Guardado, Jorge Rios

**Affiliations:** 1 Internal Medicine, Mount Carmel Health System, Grove City, USA; 2 Hematology and Oncology, Zangmeister Cancer Center, Columbus, USA

**Keywords:** anca associated vasculitis, chondritis, diagnostic delay, macrocytic anemia, vexas syndrome

## Abstract

Vacuoles, E1 enzyme, X-linked, autoinflammatory, somatic (VEXAS) syndrome is a recently described adult-onset autoinflammatory disorder caused by somatic mutations in the UBA1 gene, characterized by systemic inflammation and hematologic abnormalities. Due to its broad and overlapping clinical manifestations, VEXAS is frequently misdiagnosed as other rheumatologic or hematologic conditions, leading to delays in diagnosis.

We present the case of a 67-year-old man initially diagnosed with antineutrophil cytoplasmic antibody (ANCA)-associated vasculitis who developed progressive multisystem inflammatory disease, including migratory inflammatory arthritis, chondritis, scleritis, constitutional symptoms, and persistent macrocytic anemia. Despite treatment with corticosteroids and rituximab, his symptoms persisted. Bone marrow biopsy demonstrated cytoplasmic vacuolization in myeloid and erythroid precursors, and subsequent next-generation sequencing identified a somatic UBA1 mutation, confirming the diagnosis of VEXAS syndrome. His clinical course was further notable for the development of neutrophilic dermatosis consistent with Sweet’s syndrome.

This case highlights the diagnostic complexity of VEXAS syndrome and underscores the importance of recognizing characteristic clinical patterns, including macrocytic anemia, chondritis, and dermatologic manifestations, particularly in older male patients with refractory inflammatory disease. Early consideration of VEXAS and timely molecular testing may reduce diagnostic delay and facilitate more targeted management of this emerging hematoinflammatory disorder.

## Introduction

Vacuoles, E1 enzyme, X-linked, autoinflammatory, somatic (VEXAS) syndrome is a recently described adult-onset autoinflammatory disorder caused by somatic mutations in UBA1, a gene encoding the major E1 ubiquitin-activating enzyme required for initiation of cellular ubiquitination [1]. Pathogenic UBA1 mutations impair ubiquitination and protein degradation pathways, resulting in activation of innate immune signaling, increased inflammatory cytokine production, and myeloid-driven systemic inflammation [1,2]. First identified in 2020, VEXAS is now recognized as a hematoinflammatory syndrome bridging rheumatologic, dermatologic, and hematologic disease processes [1,2]. The syndrome predominantly affects older men and is characterized by systemic inflammation, macrocytic anemia, cytopenias, and bone marrow findings of cytoplasmic vacuolization in myeloid and erythroid precursors [1,3-5].

Clinical manifestations are heterogeneous and often include chondritis, pulmonary involvement, ocular inflammation, inflammatory arthritis, constitutional symptoms, and cutaneous disease [1,4-7]. Hematologic abnormalities frequently include myelodysplastic features, clonal hematopoiesis, and plasma cell dyscrasias [4-7]. Among dermatologic manifestations, neutrophilic dermatoses such as Sweet’s syndrome have been increasingly recognized and may represent an early or evolving feature of disease [4,7,8].

Despite increasing awareness, VEXAS remains underdiagnosed due to its significant overlap with more common conditions, including ANCA-associated vasculitis, relapsing polychondritis, and myelodysplastic syndromes [2,4-7]. Definitive diagnosis requires molecular confirmation of somatic UBA1 mutations, often after prolonged evaluation across multiple specialties [1,5]. We report a case of VEXAS syndrome initially treated as ANCA-associated vasculitis, with subsequent development of neutrophilic dermatosis, highlighting the diagnostic delay and the importance of recognizing characteristic clinical patterns.

## Case presentation

A 67-year-old man with a history of hypertension, hyperlipidemia, osteoarthritis, microscopic polyangiitis, melanoma, and squamous cell carcinoma of the scalp was referred for hematologic evaluation in the setting of progressive macrocytic anemia and recurrent inflammatory arthralgias. His symptoms began in the fall of 2023 with bilateral knee pain that initially self-resolved, followed by the development of migratory inflammatory arthralgias involving the hands and feet, associated with swelling and episodic synovitis.

Over time, he developed additional systemic manifestations including fatigue, anorexia, unintentional weight loss of approximately 10-15 pounds, dyspnea on exertion, and left ear swelling consistent with chondral inflammation. He was also diagnosed with scleritis by ophthalmology and reported sinus symptoms and jaw pain. Given his history of melanoma and squamous cell carcinoma of the scalp, a malignancy-associated or paraneoplastic inflammatory process was considered as part of the differential diagnosis. However, the combination of recurrent chondral inflammation, ocular involvement, systemic symptoms, macrocytic anemia/cytopenias, and subsequent bone marrow findings of cytoplasmic vacuolization raised concern for VEXAS syndrome; molecular testing later confirmed a somatic UBA1 mutation.

He was evaluated by rheumatology in April 2024. Laboratory studies demonstrated positive cytoplasmic antineutrophil cytoplasmic antibody (C-ANCA) (1:160) with elevated myeloperoxidase levels, and he was diagnosed with microscopic polyangiitis. He was started on prednisone 60 mg daily with partial improvement in symptoms and was subsequently treated with rituximab.

Despite therapy, the patient continued to experience persistent systemic and inflammatory symptoms, including migratory inflammatory arthralgias with episodic synovitis, fatigue, anorexia, unintentional weight loss, dyspnea on exertion, left auricular swelling consistent with chondritis, scleritis, sinus symptoms, and jaw pain. Hematology consultation was obtained due to anemia and abnormal serum free light chains. Laboratory evaluation revealed mild macrocytic anemia (hemoglobin 11.2-12.7 g/dL, mean corpuscular volume 104-110 fL) with intermittent leukopenia. Serum protein electrophoresis and immunofixation were negative. Representative peripheral blood laboratory findings from the diagnostic evaluation are summarized in Table 1.

**Table 1:** Peripheral blood laboratory findings Representative laboratory values obtained during hematologic evaluation. WBC: white blood cells; RBC: red blood cells; MCV: mean corpuscular volume; MCHC: mean corpuscular hemoglobin concentration; RDW: red cell distribution width

Parameter	Value	Reference Range
WBC	5.0 K/µL	4.0-11.0 K/µL
RBC	3.50 M/µL	4.5-5.9 M/µL
Hemoglobin	12.1 g/dL	13.5-17.5 g/dL
Hematocrit	36%	41-53%
MCV	104 fL	80-100 fL
MCHC	33.2 g/dL	32-36 g/dL
RDW	13%	11.5-14.5%
Platelets	224 K/µL	150-400 K/µL
Granulocytes	64%	40-70%
Lymphocytes	0.275	20-40%
Monocytes	7.60%	2-8%
Eosinophils	0.20%	0-5%
Basophils	0.20%	0-1%

A bone marrow biopsy demonstrated a normocellular marrow with trilineage hematopoiesis, minimal dyserythropoiesis, and no increase in blasts (<2%). Iron stores were adequate, with approximately 5% ring sideroblasts, and serum ferritin was elevated at 356 ng/mL. Plasma cells were not increased and were polyclonal. Notably, cytoplasmic vacuolization was observed in erythroid and myeloid precursors. Cytogenetic analysis revealed a normal male karyotype, and flow cytometry showed no abnormal hematolymphoid population. Bone marrow aspirate differential findings are summarized in Table 2.

**Table 2:** Bone marrow aspirate differential Bone marrow aspirate differential obtained during diagnostic evaluation. M:E: myeloid-to-erythroid ratio; Reference ranges may vary by laboratory and clinical context.

Parameter	Value	Typical Reference Range
Blasts	1%	<5%
Promyelocytes	0%	1-5%
Myelo/meta/band/segs	66%	50-70%
Eosinophils and precursors	0%	1-6%
Basophils and precursors	0%	<1%
Monocytes	6%	0-4%
Lymphocytes	6%	5-15%
Plasma cells	1%	<5%
Normoblasts (erythroid precursors)	20%	15-30%
M:E ratio	3.65	2:1-4:1

Next-generation sequencing identified a somatic UBA1 mutation (p.Met41Leu) with a variant allele frequency of 55.44%, along with additional mutations in ARID1B, ATM, and ROS1. These findings were diagnostic of VEXAS syndrome, although no overt morphologic evidence of myelodysplastic syndrome was present at that time.

In August 2025, the patient developed diffuse erythematous papules and pustules involving the arms, chest, back, scalp, and lower extremities. Dermatologic evaluation was consistent with neutrophilic dermatosis, and a diagnosis of Sweet’s syndrome was made.

Treatment was subsequently directed toward the underlying inflammatory disorder. Tocilizumab was initiated given persistent systemic inflammatory symptoms despite corticosteroid and rituximab therapy, as well as emerging evidence supporting interleukin-6 inhibition in VEXAS syndrome. Prednisone was tapered to 5 mg daily. Colchicine was later added for management of cutaneous manifestations. The patient reported significant improvement in fatigue and systemic symptoms while receiving tocilizumab. Notably, a temporary interruption in therapy due to Mohs procedures for squamous cell carcinoma of the scalp resulted in clinical worsening, followed by improvement upon resumption of treatment. He remains under close monitoring for disease progression and potential hematologic complications.

## Discussion

This case highlights the diagnostic complexity of VEXAS syndrome and its ability to mimic ANCA-associated vasculitis, resulting in delayed recognition despite the presence of characteristic features. Our patient initially presented with multisystem inflammatory manifestations including inflammatory arthritis, chondritis, ocular inflammation, constitutional symptoms, and positive ANCA serologies, leading to an initial diagnosis of microscopic polyangiitis. However, VEXAS has been increasingly recognized to present with overlapping rheumatologic, dermatologic, and hematologic features, often leading to initial misclassification [2,4-7].

Our patient’s persistent macrocytic anemia, constitutional symptoms, and characteristic bone marrow findings ultimately raised suspicion for VEXAS syndrome. This case emphasizes the importance of considering VEXAS in older male patients with refractory multisystem inflammatory disease accompanied by unexplained hematologic abnormalities, particularly macrocytic anemia, cytopenias, or cytoplasmic vacuolization in myeloid and erythroid precursors [2,5].

A critical aspect of this case is the presence of a specific constellation of findings rather than nonspecific “systemic inflammation.” These included migratory inflammatory arthritis, chondral ear inflammation, scleritis, constitutional symptoms with significant weight loss, dyspnea on exertion, and persistent macrocytic anemia. This combination is highly consistent with the expanding clinical spectrum of VEXAS and should prompt early consideration of the diagnosis, particularly in older male patients [4-7].

Hematologic findings were particularly informative in this case. The patient demonstrated persistent macrocytic anemia and bone marrow findings of cytoplasmic vacuolization in myeloid and erythroid precursors. Although no single physical examination finding is pathognomonic, the coexistence of recurrent chondritis, neutrophilic dermatosis, ocular inflammation, constitutional symptoms, and unexplained cytopenias should raise suspicion for VEXAS syndrome and prompt consideration of UBA1 molecular testing [2,5].

The identification of a somatic UBA1 mutation confirmed the diagnosis. Additionally, the presence of co-occurring mutations in ARID1B, ATM, and ROS1 highlights the emerging understanding of VEXAS as a disorder associated with clonal hematopoiesis. While most studies have focused on mutations in genes such as DNMT3A and TET2, the broader clonal landscape remains incompletely characterized, and these findings may have implications for disease progression and hematologic risk [4-7].

The development of neutrophilic dermatosis later in the disease course further supports the diagnosis. Cutaneous manifestations, particularly Sweet’s syndrome, are well described in VEXAS and may represent either an early or late feature of disease [4,7,8]. In this patient, the delayed appearance of dermatologic findings underscores the evolving and multisystem nature of the syndrome.

Management of VEXAS remains challenging, as no standardized treatment approach exists. Many patients exhibit steroid dependence, and conventional immunosuppressive therapies have variable efficacy [2]. Targeted therapies, including interleukin-6 inhibitors and Janus kinase inhibitors, have shown promise in recent studies [2,3]. In this case, treatment with tocilizumab resulted in clinical improvement, consistent with a cytokine-driven inflammatory process.

Overall, this case underscores the importance of early recognition of VEXAS syndrome in patients with unexplained multisystem inflammation, particularly when features include macrocytic anemia, chondritis, neutrophilic dermatosis, and refractory inflammatory symptoms. Early consideration of VEXAS and timely molecular testing may reduce diagnostic delay and improve patient outcomes.

## Conclusions

VEXAS syndrome is an emerging hemato-inflammatory disorder that can closely mimic ANCA-associated vasculitis and other multisystem inflammatory conditions. This case demonstrates the importance of recognizing specific clinical patterns, including macrocytic anemia, chondritis, and neutrophilic dermatosis, in order to prompt early diagnostic evaluation. Increased awareness and timely molecular testing are essential to reduce diagnostic delay and guide appropriate management.
